# Prevalence and prognostic significance of extrarenal manifestations in patients with lupus nephritis: A retrospective cohort study

**DOI:** 10.1371/journal.pone.0362170

**Published:** 2026-11-10

**Authors:** Young-Eun Kim, Soo Min Ahn, Ji Seon Oh, Yong‑Gil Kim, Chang‑Keun Lee, Bin Yoo, Seokchan Hong

**Affiliations:** 1 Department of Rheumatology, Asan Medical Centre, University of Ulsan College of Medicine, Seoul, Republic of Korea; 2 Information Medicine, Big Data Research Centre, Asan Medical Centre, Seoul, Republic of Korea; Prime Hospital LLC, UNITED ARAB EMIRATES

## Abstract

**Objectives:**

To evaluate the prevalence and prognostic implications of extrarenal involvement in lupus nephritis (LN), with emphasis on treatment response and the risk of future renal and extrarenal flares.

**Methods:**

We retrospectively reviewed patients newly diagnosed with LN between January 2006 and December 2022 and categorized them according to the presence of extrarenal involvement at diagnosis. Renal response and remission at 12 months, defined according to the Definition of Remission in SLE (DORIS), as well as the incidence of renal and extrarenal flares during follow-up, were compared between groups. Factors associated with DORIS remission and renal response were assessed using multivariable logistic regression analyses.

**Results:**

Among 348 patients with LN, 156 (44.8%) had extrarenal involvement at diagnosis, most commonly hematological manifestations (59.0%). Rates of complete renal response (CRR) and DORIS remission at 12 months were comparable between patients with and without extrarenal involvement (CRR: 57.1% vs. 46.9%, *p* = 0.06; DORIS remission: 21.8% vs. 29.7%, *p* = 0.09). Over a median follow-up of 79.6 months, the incidence of renal flares (28.8% vs. 26.0%, *p* = 0.56) and extrarenal flares (26.3% vs. 24.5%, *p* = 0.74) did not differ significantly between groups. In multivariable analyses, baseline extrarenal involvement was not associated with DORIS remission (odds ratio [OR], 1.307; 95% confidence interval [CI]: 0.779–2.193; *p* = 0.310) or renal response (OR, 0.974; 95% CI: 0.564–1.682; *p* = 0.925).

**Conclusions:**

Extrarenal involvement is present in nearly half of patients at LN diagnosis, however, it does not appear to influence renal response, DORIS remission, or the long-term risk of renal or extrarenal flares.

## Introduction

Systemic lupus erythematosus (SLE) is a chronic systemic autoimmune disorder characterised by heterogeneous clinical manifestations affecting multiple organ systems [1]. Lupus nephritis (LN) is one of the most severe complications of SLE and remains a major cause of morbidity due to its high prevalence and the risk of progression to chronic kidney disease (CKD) and end-stage kidney failure [2]. In addition to renal involvement, patients with SLE frequently develop extrarenal manifestations, including hematologic, mucocutaneous, musculoskeletal, neuropsychiatric, and cardiopulmonary involvement.

Current treatment strategies for LN aim to induce a renal response, prevent renal and systemic flares, minimize glucocorticoid exposure, and preserve long-term kidney function. Glucocorticoids, administered in combination with immunosuppressive agents such as mycophenolate mofetil (MMF) or cyclophosphamide (CYP), remain the cornerstone of induction therapy, while add-on therapies, including belimumab and calcineurin inhibitors, may be considered in selected patients according to disease severity and prognostic factors [3–5]. Achieving a favourable renal response is a primary therapeutic goal in LN [6–8]. However, the clinical relevance of extrarenal involvement at the time of LN diagnosis remains unclear. Previous studies have shown that extrarenal manifestations in patients with LN are associated with higher overall disease activity and poorer health-related quality of life [9,10]. In contrast, a systematic review reported inconsistent associations between systemic manifestations and clinical outcomes across studies [11].

Emerging evidence suggests that achieving systemic disease control alongside renal response may provide additional prognostic benefit in LN, particularly by reducing the risk of subsequent renal relapse [12,13]. However, although extrarenal involvement may influence treatment intensity, glucocorticoid exposure, and follow-up strategies, it remains unclear whether baseline extrarenal involvement independently predicts treatment response and remission beyond established renal, serological, histological, and treatment-related factors. Therefore, the prognostic implications of extrarenal involvement in LN require further clarification.

In addition, the clinical course of SLE is highly variable, characterized by periods of remission and relapse [14–16]. Disease flares are clinically significant events associated with increased morbidity, cumulative organ damage, and adverse long-term outcomes [17]. Nevertheless, whether extrarenal involvement at the time of LN diagnosis independently influences the subsequent renal response or the achievement of systemic remission remains unclear. Therefore, this study aimed to investigate the prevalence and clinical significance of extrarenal involvement at the time of LN diagnosis. Specifically, we evaluated its associations with treatment response, achievement of the Definition of Remission in SLE (DORIS) remission, and the risk of subsequent renal and extrarenal flares in a large cohort of patients with newly diagnosed LN.

## Materials and methods

### Study population

The medical records of patients newly diagnosed with LN between January 2006 and December 2022 were retrospectively reviewed. Patients who underwent kidney biopsy were diagnosed according to the histological criteria defined by the International Society of Nephrology/Renal Pathology Society (ISN/RPS) 2018 classification [18]. Patients who did not undergo kidney biopsy were included if they fulfilled the 2019 SLE classification criteria [19] and had clinical features consistent with LN, suggestive of glomerulonephritis, including proteinuria >500 mg/day and hematuria. Patients with <12 months of follow-up were excluded from the study. Patients were categorized into two groups based on the presence or absence of extrarenal involvement at the time of LN diagnosis. Extrarenal involvement was defined according to the constitutional, mucocutaneous, musculoskeletal, cardiac, respiratory, neuropsychiatric, and hematological domains of the British Isles Lupus Assessment Group index [20]. Among the manifestations of extrarenal involvement, major extrarenal organ involvement was defined as the presence of central nervous system involvement, cardiopulmonary involvement, vasculitis, fever, or hemolytic anemia, based on the major organ/system activity domains included in the Lupus Low Disease Activity State (LLDAS) definition [21]. The study protocol was approved by the Institutional Review Board (IRB) of Asan Medical Centre, Seoul, South Korea (IRB No. 2023−0804), which waived the requirement for informed consent owing to the retrospective nature of the study. For this retrospective study, the medical records and data were accessed for research purposes from July 1, 2023 to March 31, 2024.

### Data collection

Clinical data were extracted from patients’ medical records and included demographic characteristics (age, sex, and body mass index). Laboratory parameters included serum anti-double-stranded DNA (anti-dsDNA) antibody levels and complement concentrations. Serum anti-dsDNA antibody levels and complement C3 and C4 concentrations were measured as part of routine clinical care at the central laboratory of our center. Anti-dsDNA antibody levels were measured using an immunoradiometric assay (Anti-ds DNA kit; Trinity Biotech, Ireland), and serum complement C3 and C4 concentrations were measured using a latex-enhanced immunoturbidimetric assay (Atellica CH Complement, Siemens Healthcare Diagnostics, Germany). The estimated glomerular filtration rate (eGFR) was calculated using the Chronic Kidney Disease Epidemiology Collaboration equation. Disease activity at the time of LN diagnosis was assessed using the SLE Disease Activity Index 2000 (SLEDAI-2K) [22], and nonrenal disease activity was evaluated using the nonrenal SLEDAI-2K score. Histologic class of LN was determined according to the ISN/RPS 2018 classification [18]. Treatment-related variables included the use of hydroxychloroquine; induction and maintenance immunosuppressive therapies, including CYP, MMF, calcineurin inhibitors, rituximab, and belimumab; the initial daily dose of oral glucocorticoids prescribed after LN diagnosis; the average oral glucocorticoid dose during follow-up; and the use of intravenous glucocorticoid pulse therapy as part of the initial treatment for LN. Patients with an inadequate response to the initial regimen who subsequently received an alternative induction regimen (CYP or MMF) were classified separately as having undergone re-induction therapy.

### Treatment outcomes

During each follow-up visit, SLEDAI-2K scores and flare status were assessed. An extrarenal flare was defined as an increase of three or more points in the nonrenal SLEDAI score, followed by subsequent improvement after initiation of corticosteroid therapy or an increase in corticosteroid dose [23,24]. Clinical characteristics and medications, including immunosuppressive agents, during each flare episode were recorded. A renal flare was defined as an increase in proteinuria and/or serum creatinine level, the presence of active urinary sediment, or a reduction in creatinine clearance, consistent with active renal disease [25,26]. Specifically, renal flare included an increase in serum creatinine level of ≥30% compared with the previous measurement, accompanied by nephritic urinary sediment, or an increase in proteinuria without concomitant rise in serum creatinine levels. Proteinuria was considered increased if it rose by ≥2 g/day when baseline levels were <3.5 g/day or if it doubled when baseline proteinuria was within the nephrotic range.

Renal response was evaluated at 12 months after treatment initiation according to following criteria used in the AURORA 1 trial; Complete renal response (CRR) was defined as normal renal function (eGFR ≥ 60 ml/min/1.73 m^2^ or no decrease >20% from baseline) and proteinuria ≤0.5 g/day, together with a daily prednisone dose ≤10 mg [4]. Partial renal response (PRR) was defined as ≥50% reduction in proteinuria from baseline. Nonresponse was defined as failure to meet criteria for either CRR or PRR. CKD was defined as an eGFR < 60 mL/min/1.73 m^2^ persisting for >3 months. End-stage kidney disease (ESKD) was defined as the requirement for dialysis for >3 months or the need for kidney transplantation.

DORIS remission on therapy was defined as a clinical SLEDAI-2K score of 0 in the absence of renal activity and a daily prednisone dose ≤5 mg, irrespective of ongoing immunosuppressive or biologic therapy [27].

### Statistical analysis

Parametric continuous variables are presented as means with standard deviations, whereas nonparametric continuous variables are presented as medians and interquartile ranges (IQRs). Continuous variables were compared using Student’s t-test or Mann–Whitney U test, as appropriate. Categorical variables were compared using the χ^2^ test or Fisher’s exact test, as appropriate. Time-to-event data for renal and extrarenal flares were analysed using Kaplan–Meier survival curves and compared between groups using the log-rank test. Multivariable Cox proportional hazards regression analyses were performed to estimate hazard ratios with 95% confidence intervals (CIs). For the time-to-event analyses, follow-up began on the date of LN diagnosis. Renal and extrarenal flares were analyzed separately, with the first renal or extrarenal flare after LN diagnosis considered the respective event. Patients without the outcome of interest were censored at the end of available clinical follow-up. Univariable and multivariable logistic regression analyses were performed to identify factors associated with DORIS remission and renal response (CRR or PRR) at 12 months. To evaluate the independent association between baseline extrarenal involvement and 12-month outcomes, multivariable logistic regression models were constructed using clinically relevant covariates. The same primary adjustment set was used for the multivariable logistic regression analyses of DORIS remission and renal response. The common adjustment set included age, sex, BMI, year of LN diagnosis, baseline eGFR, and baseline extrarenal involvement. Baseline extrarenal involvement was the primary exposure of interest. These covariates were selected independently of the results of the univariable analyses. In additional exploratory analyses, variables associated with each outcome in the univariable analyses and considered clinically relevant were added to the common adjustment model. Baseline SLEDAI-2K score and immunosuppressive drug use were added to the exploratory model for DORIS remission, whereas nephrotic-range proteinuria and hydroxychloroquine use were added to the exploratory model for renal response. A focused set of covariates was selected to minimize statistical redundancy and potential collinearity, with one representative variable chosen when variables represented overlapping clinical constructs. Baseline extrarenal involvement was included in all multivariable models as the primary exposure. Individual extrarenal organ domains were evaluated separately in univariable analyses but not simultaneously with overall extrarenal involvement in the multivariable models. To account for potential treatment-era effects over the study period, the year of LN diagnosis was included as an additional covariate in the multivariable regression models. Multivariable Cox proportional hazards models were also used to evaluate the association between baseline extrarenal involvement and subsequent renal and extrarenal flares after adjustment for clinically relevant baseline characteristics and initial treatment intensity. Results are expressed as odds ratios (ORs) with 95% CIs. A two-sided p-value <0.05 was considered statistically significant.

## Results

### Baseline characteristics of the study cohort

This retrospective study included 348 patients newly diagnosed with LN between January 2006 and December 2022. During the study period, 501 patients with newly diagnosed LN were initially screened. After excluding patients with <12 months of follow-up (n = 91), or missing laboratory data or extrarenal assessment (n = 62), 348 patients were included in the final analysis (Fig 1). Of these, 156 (44.8%) patients presented with extrarenal involvement at the time of LN diagnosis, whereas 192 (55.2%) had no extrarenal manifestations (Table 1). The cohort comprised 276 (79.3%) women and 72 (20.7%) men, with a median age of 43.3 years (IQR, 31.4–54.6 years). Among the 156 patients with extrarenal involvement, hematologic manifestations were the most common, occurring in 92 patients (59.0%), with thrombocytopenia being the predominant feature. This was followed by mucocutaneous and constitutional involvement in 32 (20.5%) and 29 (18.6%) patients, respectively. A significantly higher proportion of women presented with extrarenal involvement compared with those without extrarenal involvement (133/156, 85.3% vs. 143/192, 74.5%, *p* = 0.01). The median time from SLE diagnosis to LN diagnosis was 0.0 months (IQR, 0.0–0.1) in the overall cohort and did not differ between patients with and without baseline extrarenal involvement (0.0 [0.0–0.3] vs. 0.0 [0.0–0.2] months; *p* = 0.85). Patients with extrarenal involvement exhibited significantly greater serologic activity, with lower complement C3 levels (41.0 mg/dL [IQR, 29.3–61.3 mg/dL] vs. 59.9 mg/dL [IQR, 41.9–84.1 mg/dL]; *p* < 0.01) and C4 levels (7.1 mg/dL [IQR, 4.3–11.7 mg/dL] vs. 12.6 mg/dL [IQR, 5.9–22.3 mg/dL]; *p* < 0.01), as well as higher anti-dsDNA antibody titres (33.1 IU/mL [IQR, 7.6–367.5 IU/mL] vs. 19.9 IU/mL [IQR, 5.9–149.0 IU/mL]; *p* = 0.04), than those without extrarenal involvement. Overall disease activity was also higher in patients with extrarenal involvement, as reflected by higher total SLEDAI-2K scores (13.0 [IQR, 10.0–17.0] vs. 8.0 [IQR, 6.0–12.0]; *p* < 0.01) and higher nonrenal SLEDAI-2K scores (5.0 [IQR, 4.0–7.0] vs. 4.0 [IQR, 2.0–4.0]; *p* < 0.01). Kidney pathological class distribution differed between patients with and without extrarenal involvement, with proliferative or mixed LN being more frequent among patients with extrarenal involvement.

**Table 1:** Clinical characteristics of patients newly diagnosed with lupus nephritis with and without extrarenal involvement.

	Total (n = 348)	With extrarenal involvement (n = 156, 44.8%)	Without extrarenal involvement (n = 192, 55.2%)	*P*
Age (years)	43.3 (31.4–54.6)	43.9 (30.4–56.6)	42.7 (31.8–54.9)	0.98
Female	276 (79.3)	133 (85.3)	143 (74.5)	**0.01**
BMI	21.7 (19.5–24.1)	21.0 (19.1–23.7)	22.0 (19.9–24.5)	0.07
Year of LN diagnosis				0.42
2006‒2016	206 (59.2)	96 (61.5)	110 (57.3)	
2016‒2022	142 (40.8)	60 (38.5)	82 (42.7)	
Time from SLE diagnosis	0.0 (0.0–0.1)	0.0 (0.0–0.3)	0.0 (0.0–0.2)	0.85
**Comorbidity**				
Hypertension	61 (17.5)	21 (13.5)	40 (20.8)	0.08
Diabetes mellitus	7 (2.0)	1 (0.6)	6 (3.1)	0.07
**Laboratory parameters**				
ESR (mm/h)	34.0 (17.0–55.0)	34.0 (17.5–55.0)	34.0 (17.0–55.0)	0.67
CRP (mg/dL)	0.2 (0.1–0.8)	0.4 (0.1–1.3)	0.1 (0.1–0.5)	0.29
Creatinine (mg/dL)	0.9 (0.6–1.7)	0.9 (0.6–1.4)	0.9 (0.6–2.1)	**0.02**
eGFR (mL/min/1.73 m²)	90.0 (38.8–90.0)	90.0 (43.0–90.0)	90.0 (29.0–90.0)	0.16
C3 (mg/dL)	51.3 (34.2–76.3)	41.0 (29.3–61.3)	59.9 (41.9–84.1)	**<0.01**
C4 (mg/dL)	9.1 (5.0–17.5)	7.1 (4.3–11.7)	12.6 (5.9–22.3)	**<0.01**
Anti-dsDNA (IU/mL)	25.9 (6.8–279.5)	33.1 (7.6–367.5)	19.9 (5.9–149.0)	**0.04**
Urine protein/creatinine ratio (g/g)	1.6 (0.7–4.1)	1.6 (0.8–3.7)	1.9 (0.6–4.7)	0.77
Lupus anticoagulant positivity	88 (25.3)	46 (29.5)	42 (21.9)	0.11
Anticardiolipin antibody positivity	70 (20.1)	39 (25.0)	31 (16.1)	**0.04**
Anti-β2 glycoprotein I antibody positivity	42 (12.1)	25 (16.0)	17 (8.9)	0.05
**Disease activity**				
SLEDAI	12.0 (8.0–16.0)	13.0 (10.0–17.0)	8.0 (6.0–12.0)	**<0.01**
Nonrenal SLEDAI	4.0 (2.0–5.0)	5.0 (4.0–7.0)	4.0 (2.0–4.0)	**<0.01**
Constitutional	29 (8.3)	29 (18.6)		
Hematological	92 (26.4)	92 (59.0)		
Mucocutaneous	32 (9.2)	32 (20.5)		
Musculoskeletal	12 (3.4)	12 (7.7)		
Neuropsychiatric	11 (3.2)	11 (7.1)		
**Kidney pathology** **(ISN/RPS classification)**	**n = 292**	**n = 141**	**n = 151**	**<0.01**
Proliferative/Mixed	190/44 (65.1/15.1)	98/22 (69.5/15.6)	92/22 (60.9/14.6)	
Class III/III + V	67/20	34/10	33/10	
Class IV/IV + V	123/24	64/12	59/12	
Non-proliferative	58 (19.9)	21 (14.9)	37 (24.5)	
Class I/II	1/26	1/12	0/14	
Class V/VI	30/1	8/0	22/1	
**Treatment**				
Hydroxychloroquine	275 (79.0)	131 (84.0)	144 (75.0)	**0.05**
Cyclophosphamide	153 (44.0)	77 (49.4)	76 (39.6)	0.06
Mycophenolate mofetil	195 (56.0)	79 (50.6)	116 (60.4)	0.81
Re-induction†	105 (30.2)	55 (35.3)	50 (26.0)	0.26
CNI	135 (38.8)	63 (40.4)	72 (37.5)	0.59
Rituximab	50 (14.4)	27 (17.3)	23 (12.0)	0.17
Belimumab	12 (3.4)	5 (3.2)	7 (3.6)	0.82
Average dose of GCs, mg	4.8 (2.2–7.8)	5.4 (2.5–8.2)	4.2 (1.9–7.2)	0.08
Initial GC dose, mg	30.0 (10.0–60.0)	55.0 (30.0–75.0)	20.0 (5.0–50.0)	0.18
Steroid pulse therapy	50 (14.4)	27 (17.3)	23 (12.0)	0.22
**Proliferative LN**	**n = 190**	**n = 98**	**n = 92**	
Hydroxychloroquine	187 (98.4)	96 (98.0)	91 (98.9)	0.99
Cyclophosphamide	105 (55.3)	55 (56.1)	50 (54.3)	0.87
Mycophenolate mofetil	85 (44.7)	43 (43.9)	42 (45.7)	0.95
Re-induction†	47 (24.7)	31 (31.6)	16 (17.4)	0.26
CNI	76 (40.0)	41 (41.8)	35 (38.0)	0.60
Rituximab	27 (14.2)	17 (17.3)	10 (10.9)	**0.01**
Belimumab	9 (4.7)	4 (4.1)	5 (5.4)	0.66
Average dose of GCs, mg	4.8 (2.3–7.6)	5.4 (3.1–7.9)	3.9 (1.8–6.7)	0.18

†Re-induction therapy was defined as the subsequent use of cyclophosphamide or mycophenolate mofetil following an inadequate response to the initial induction regimen

BMI: body mass index; eGFR: estimated glomerular filtration rate; dsDNA: double-stranded DNA; SLE: systemic lupus erythematosus; SLEDAI**:** SLE Disease Activity Index; ESR: erythrocyte sedimentation rate; CRP: C-reactive protein; ISN/RPS: International Society of Pathology/Renal Pathology Society; CNI: calcineurin inhibitor; GC: glucocorticoid.

**Fig 1:**
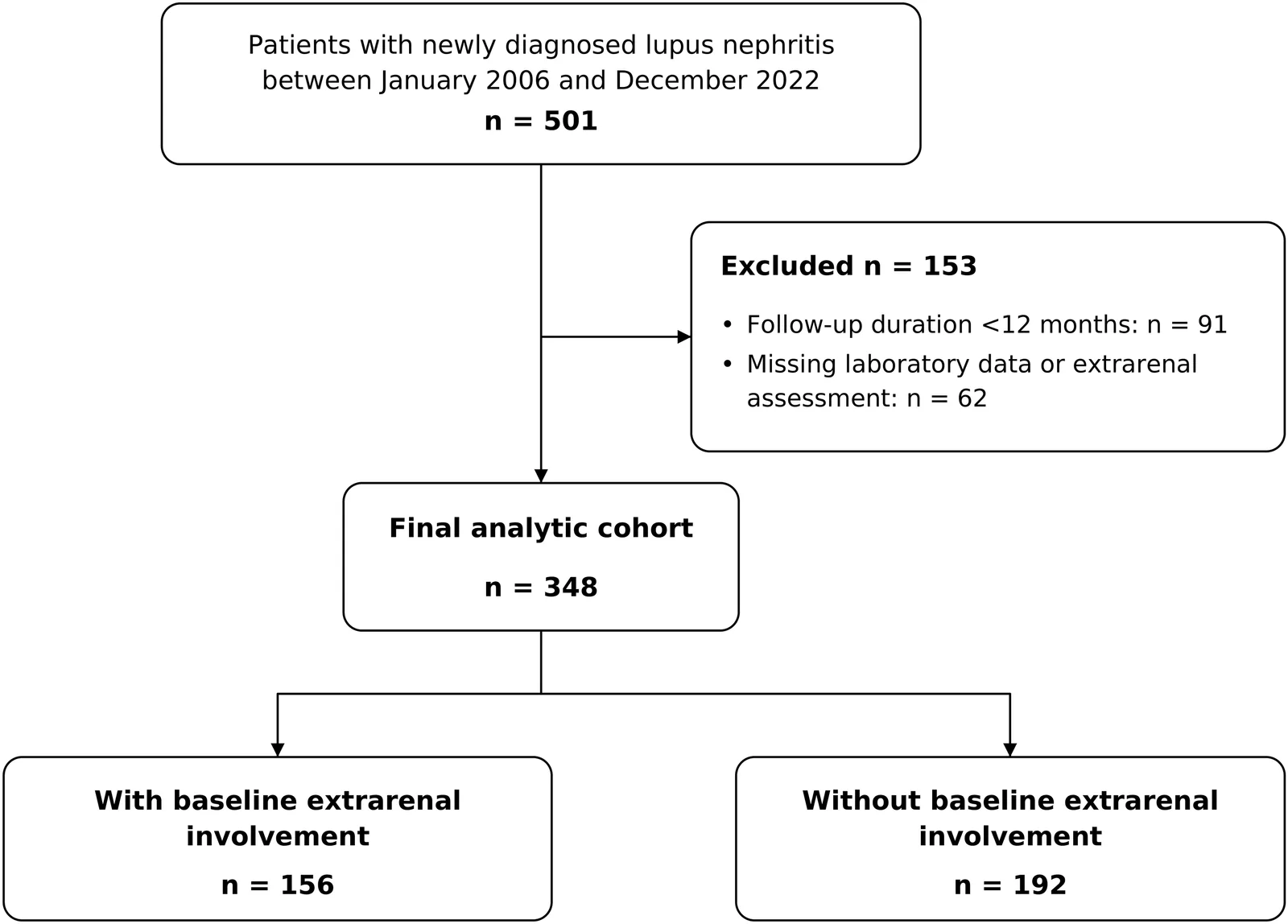
Study flow.

Data on immunosuppressive therapy after LN diagnosis were also collected. MMF was the most frequently prescribed agent (56.0%, n = 195), followed by CYP (44.0%, n = 153). The use of other immunosuppressive agents, including calcineurin inhibitors, rituximab, and belimumab, did not differ significantly between groups. The average daily oral glucocorticoid dose during follow-up and the initial oral glucocorticoid dose were comparable between patients with and without extrarenal involvement (average daily dose: 5.4 mg/day [IQR, 2.5–8.2] vs. 4.2 mg/day [IQR, 1.9–7.2]; p = 0.08; initial dose: 55.0 mg/day [IQR, 30.0–75.0] vs. 20.0 mg/day [IQR, 5.0–50.0]; p = 0.18). Among patients with proliferative LN, rituximab use was significantly more common in patients with extrarenal involvement (17.3% vs. 10.9%, *p* = 0.01).

### Clinical outcome of patients with LN with and without extrarenal involvement

We evaluated treatment response during the follow-up period. At 12 months after LN diagnosis, renal response rates did not differ significantly between patients with and without extrarenal involvement. Although CRR tended to be more frequent among patients with extrarenal involvement than among those without (57.1% [89/156] vs. 46.9% [90/192]), the difference did not reach statistical significance (*p* = 0.06; Table 2). Similarly, the rate of remission defined according to the DORIS criteria was comparable between the two groups (21.8% vs. 29.7%, *p* = 0.09).

**Table 2:** Clinical outcomes of patients with lupus nephritis with and without baseline extrarenal involvement.

	With baseline extrarenal involvement	Without baseline extrarenal involvement	*P*
***At 12 months***			
Renal response			
Complete renal response	89 (57.1)	90 (46.9)	0.06
Partial renal response	27 (17.3)	34 (17.7)	0.92
Non-response	40 (25.6)	68 (35.4)	0.05
DORIS remission at 12 months	34 (21.8)	57 (29.7)	0.09
***During total follow-up***			
Follow-up duration, median (IQR), months	95.7 (42.9–142.7)	73.6 (33.9–130.0)	0.85
Renal flare	45 (28.8)	50 (26.0)	0.56
Extrarenal flare	41 (26.3)	47 (24.5)	0.74
CKD (eGFR < 60 mL/min/1.73 m^2^)	51 (32.7)	76 (39.6)	0.19
ESKD (dialysis or transplantation)	26 (16.7)	45 (23.4)	0.12
Death	5 (3.2)	10 (5.2)	0.35

CKD: chronic kidney disease; eGFR: estimated glomerular filtration rate; ESKD: end-stage kidney disease; IQR: interquartile range; DORIS: Definition of Remission in SLE.

Over a median follow-up duration of 79.6 months (IQR, 39.2–135.2 months), both renal and extrarenal flares occurred in each group. However, the incidence of renal flares (28.8% vs. 26.0%; *p* = 0.56) and extrarenal flares (26.3% vs. 24.5%; *p* = 0.74) did not differ significantly between patients with and without baseline extrarenal involvement at LN diagnosis (Table 2; Fig 2). In the multivariable Cox proportional hazards regression analyses, baseline extrarenal involvement was not independently associated with subsequent renal flare (hazard ratio [HR], 1.121; 95% CI, 0.678‒1.853; *p* = 0.657) or extrarenal flare (HR, 0.964; 95% CI, 0.605‒1.538; *p* = 0.879) after adjustment for clinically relevant baseline characteristics and initial treatment intensity (S1 Table). Rates of progression to CKD and ESKD were also similar between groups. Among patients without extrarenal involvement at baseline, de novo extrarenal manifestations developed during follow-up (Fig 3). The most common manifestations were constitutional (30/192, 15.6%), hematologic (31/192, 16.1%), and mucocutaneous (31/192, 16.1%) involvement, whereas neuropsychiatric and cardiopulmonary involvement remained uncommon.

**Fig 2:**
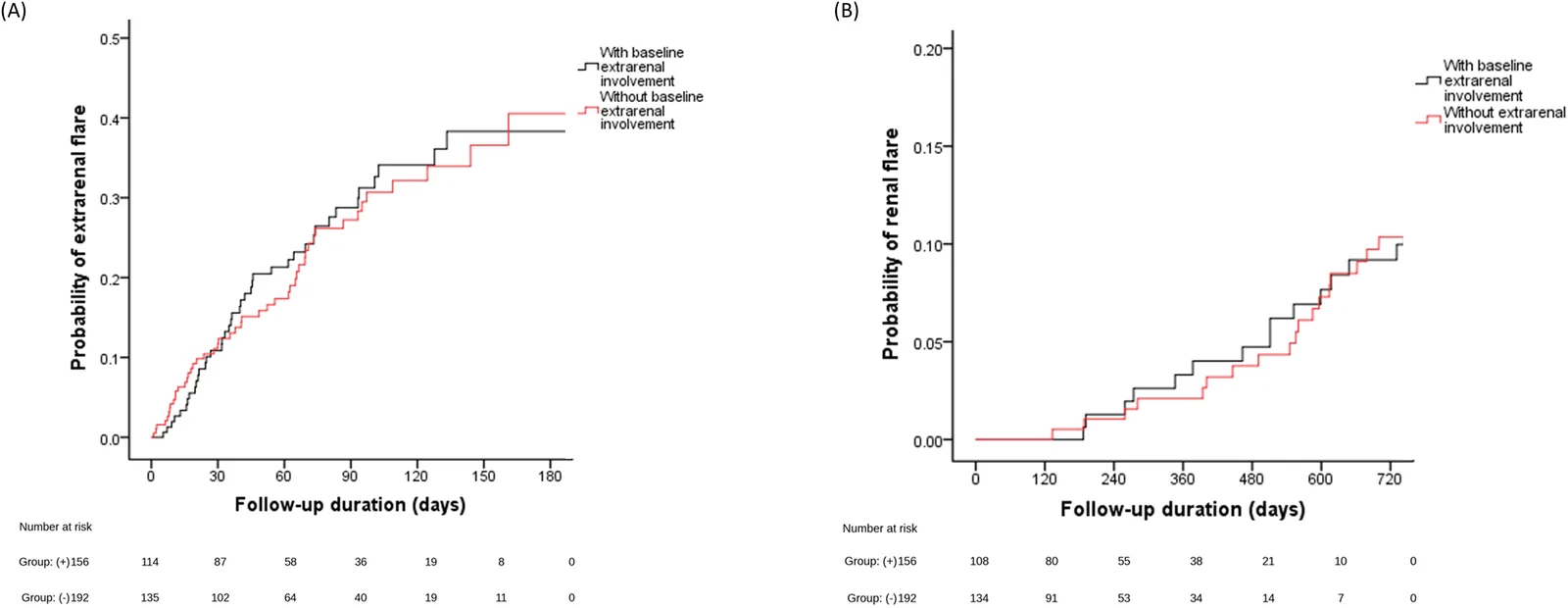
Cumulative probability of flares according to the presence of extrarenal involvement at lupus nephritis diagnosis: (A) extrarenal flare, (B) renal flare.

**Fig 3:**
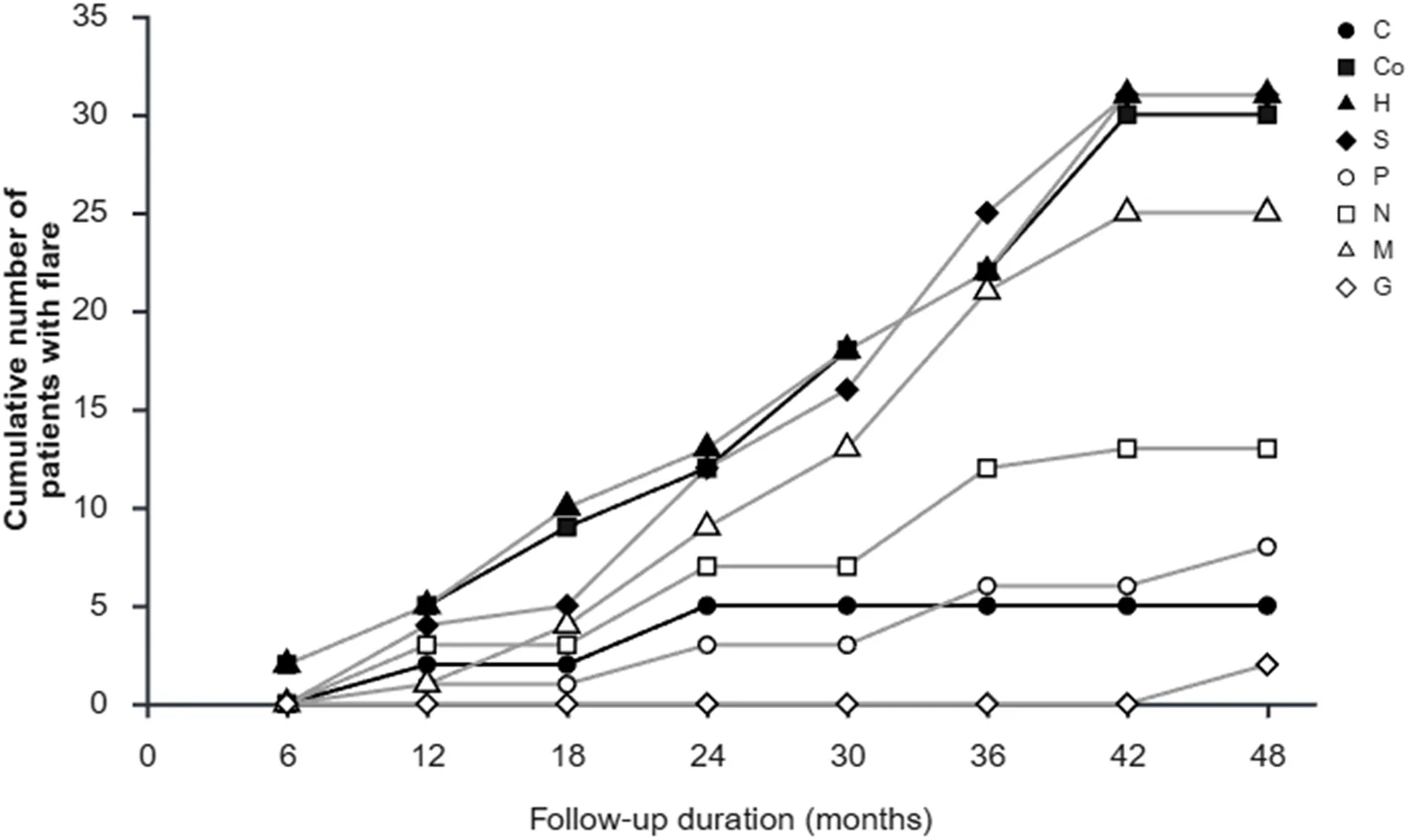
Cumulative number of patients with *de novo* extrarenal organ involvement during follow-up in patients without extrarenal involvement at baseline. C, cardiac; Co, constitutional; H, hematologic; S, mucocutaneous; P, pulmonary; N, neuropsychiatric; M, musculoskeletal; G, gastrointestinal.

### Influence of extrarenal involvement on DORIS remission and renal response

We next evaluated baseline factors, including extrarenal manifestations, associated with achievement of DORIS remission at 12 months after LN diagnosis (Table 3). In multivariable analyses, female sex was associated with a higher likelihood of achieving DORIS remission at 12 months (OR, 2.415; 95% CI, 1.343–4.341; *p* = 0.003). In contrast, baseline extrarenal involvement—either evaluated as a composite variable or stratified by individual domains (mucocutaneous, constitutional, hematologic, musculoskeletal, or neuropsychiatric)—was not significantly associated with achievement of DORIS remission (any extrarenal involvement: OR, 1.307; 95% CI, 0.779–2.193; *p* = 0.310). In addition, LN diagnosis after 2016 was independently associated with a higher likelihood of achieving DORIS remission at 12 months (OR, 2.080; 95% CI, 1.248–3.468; *p* = 0.005).

**Table 3:** Factors associated with DORIS remission at 12 months.

	Univariable		Multivariable	
	OR (95% CI)	*P*	OR (95% CI)	*P*
Age, per 10-year increase	1.151 (0.613–2.162)	0.662	1.039 (0.877–1.231)	0.660
BMI	**1.070 (1.001–1.145)**	**0.048**	1.045 (0.973‒1.123)	0.227
Baseline eGFR	0.999 (0.992–1.007)	0.889	0.999 (0.992–1.008)	0.954
Female	**2.228 (1.279–3.879)**	**0.005**	**2.415 (1.343–4.341)**	**0.003**
Year of diagnosis				
2006‒2016	–			
2016‒2022	**1.822 (1.124–2.952)**	**0.015**	**2.080 (1.248–3.468)**	**0.005**
At baseline				
Extrarenal involvement	1.515 (0.928–2.474)	0.097	1.307 (0.779‒2.193)	0.310
Mucocutaneous	0.773 (0.323–1.854)	0.565		
Constitutional	0.718 (0.283–1.824)	0.486		
Respiratory	0.227 (0.029–1.770)	0.157		
Cardiac	2.197 (0.741–6.514)	0.156		
Hematological	0.786 (0.449–1.375)	0.398		
SLEDAI (per 1-point increase)	**0.929 (0.886–0.973)**	**0.002**		
Proliferative type LN	0.635 (0.390–1.036)	0.069		
Nephrotic-range proteinuria	1.092 (0.645–1.849)	0.743		
aPL positivity†	1.202 (0.739–1.957)	0.458		
Medication				
Hydroxychloroquine	0.963 (0.533–1.740)	0.901		
IS drugs‡	**0.489 (0.301–0.795)**	**0.004**		
Belimumab	1.782 (0.370–8.587)	0.471		
Initial dose of GCs	0.999 (0.998–1.001)	0.313		

†Antiphospholipid antibody (aPL) positivity was defined as positivity for at least one of the following: lupus anticoagulant, anti-β2 glycoprotein I antibody, or anticardiolipin antibody. ‡IS drugs include cyclophosphamide, mycophenolate mofetil, rituximab, and calcineurin inhibitors

LN: lupus nephritis; OR: odds ratio; IS drugs: immunosuppressive drugs; CI: confidence interval; SLEDAI**:** SLE Disease Activity Index; DORIS: Definition of Remission in SLE; BMI: body mass index; GCs: glucocorticoids

In exploratory analyses, the use of immunosuppressive agents was inversely associated with remission, although the association was not significant (OR, 0.609; 95% CI, 0.356–1.041; *p* = 0.070). Higher baseline SLEDAI-2K scores were also associated with a lower likelihood of remission (per 1-point increase, OR, 0.954; 95% CI, 0.902–0.999; *p* = 0.048) (S2 Table).

Factors associated with renal response at 12 months after LN diagnosis were then examined (**Table 4**). Older age was associated with a lower likelihood of achieving renal response at 12 months, although this association did not reach statistical significance (OR, 0.930 per 10-year increase; 95% CI, 0.775–1.115; *p* = 0.430). Notably, the presence of extrarenal involvement at baseline, including individual organ domains, was not a significant predictor of renal response (any extrarenal involvement: OR, 0.974; 95% CI, 0.564–1.682; *p* = 0.925). Furthermore, even when extrarenal involvement was restricted to major extrarenal organ involvement, no significant association was observed with either DORIS remission or renal response at 12 months (S3 and S4 Tables).

**Table 4:** Factors associated with renal response at 12 months.

	Univariable		Multivariable	
	OR (95% CI)	*P*	OR (95% CI)	*P*
Age, per 10-year increase	**0.507 (0.282–0.911)**	**0.023**	0.930 (0.775–1.115)	0.430
BMI	0.991 (0.913–1.076)	0.831	0.983 (0.910‒1.063)	0.673
Baseline eGFR	**1.027 (1.019‒1.035)**	**<0.001**	**1.026 (1.018‒1.035)**	**<0.001**
Female	1.408 (0.800–2.479)	0.235	1.431 (0.744‒2.752)	0.283
Year of diagnosis				
2006‒2016	–			
2016‒2022	1.326 (0.810–2.170)	0.262	1.192 (0.689–2.062)	0.530
At baseline				
Extrarenal involvement	0.823 (0.508–1.333)	0.429	0.974 (0.564–1.682)	0.925
Mucocutaneous	1.071 (0.456–2.515)	0.875		
Constitutional	0.910 (0.376–2.202)	0.834		
Respiratory	0.405 (0.132–1.244)	0.114		
Cardiac	1.424 (0.380–5.341)	0.600		
Hematological	0.965 (0.552–1.685)	0.900		
SLEDAI (per 1-point increase)	1.015 (0.959–1.074)	0.608		
Proliferative type LN	1.364 (0.846–2.200)	0.202		
Nephrotic-range proteinuria	**0.509 (0.280–0.924)**	**0.027**		
aPL positivity†	1.325 (0.804‒2.183)	0.710		
Medication				
Hydroxychloroquine	**4.630 (2.655–8.076)**	**<0.001**		
IS drugs‡	0.967 (0.660–1.560)	0.891		
Belimumab	3.777 (0.474–30.102)	0.210		
Initial dose of GCs	0.999 (0.998–1.000)	0.086		

Renal response was defined as achievement of either complete renal response or partial renal response at 12 months after LN diagnosis. †Antiphospholipid antibody (aPL) positivity was defined as positivity for at least one of the following: lupus anticoagulant, anti-β2 glycoprotein I antibody, or anticardiolipin antibody. ‡IS drugs include cyclophosphamide, mycophenolate mofetil, rituximab, and calcineurin inhibitors

LN: lupus nephritis; OR: odds ratio; IS drugs: immunosuppressive drugs; CI: confidence interval; SLEDAI**:** SLE Disease Activity Index; BMI: body mass index; eGFR: estimated glomerular filtration rate; aPL: antiphospholipid antibody; GCs: glucocorticoids

Exploratory analyses showed that histologic class was not significantly associated with renal response; however, nephrotic-range proteinuria was associated with a reduced likelihood of achieving renal response (OR, 0.811; 95% CI, 0.432–0.974; *p* = 0.045). Use of hydroxychloroquine was strongly associated with renal response (OR, 4.294; 95% CI, 2.411–7.649; *p* < 0.001) (S5 Table).

## Discussion

In this retrospective study of 348 patients with newly diagnosed LN, nearly half of the patients (44.8%) presented with extrarenal involvement at diagnosis, most commonly hematologic manifestations, underscoring the systemic nature of SLE. Although extrarenal involvement was associated with higher baseline disease activity and more pronounced serologic abnormalities, it did not independently predict 12-month renal response, achievement of DORIS remission, or long-term risks of renal or extrarenal flares.

The management of LN has evolved toward individualized strategies that aim to induce renal response, prevent flares, minimize glucocorticoid exposure, and preserve long-term kidney function. Current treatment approaches typically include glucocorticoids combined with MMF or CYP as the cornerstone of induction therapy, with adjunctive therapies, such as belimumab or calcineurin inhibitors, considered in selected patients according to disease severity and prognostic factors [6,8]. Although patients with extrarenal involvement had lower complement levels and higher anti-dsDNA antibody titers, indicating greater systemic immune activation at LN diagnosis, these serological differences were not associated with worse renal response, DORIS remission, or long-term flare risk. The reported prevalence and pattern of extrarenal involvement in patients with LN vary widely across studies, likely reflecting differences in study populations, disease definitions, and timing of assessment. A previous study of 299 patients with LN reported hematologic abnormalities as the most frequent extrarenal manifestation (87.3%), followed by arthritis (69.6%) and mucocutaneous symptoms (62.9%) [28]. Conversely, an analysis of 1,604 patients with LN in Iran found that 80% of patients presented with extrarenal involvement, with constitutional symptoms being the most common manifestations [29]. In contrast, a multiethnic cohort of 181 patients reported a 41% prevalence of extrarenal manifestations, most frequently mucocutaneous involvement [9]. In our cohort of patients with newly diagnosed LN, the prevalence of extrarenal involvement was 44.8%, with a predominance of hematologic manifestations. Our findings contribute to the existing literature by characterizing the burden and spectrum of extrarenal involvement at the time of LN diagnosis in a large, well-defined cohort.

Patients with extrarenal involvement in the present study exhibited significantly higher baseline disease activity, as assessed by both total and nonrenal SLEDAI-2K scores. Nonetheless, baseline extrarenal involvement did not independently predict achievement of DORIS remission at 12 months. Instead, remission was more closely linked to markers of overall disease severity and treatment-related factors, including female sex, baseline SLEDAI-2K score, and use of immunosuppressive agents. Previous studies have suggested that extensive disease activity may cluster with more severe systemic involvement and lower rates of DORIS remission [30,31]. However, the independent correlation between systematically defined extrarenal manifestations—whether evaluated as a composite measure or by individual domains—and DORIS remission remains insufficiently characterized. Consistent with prior reports indicating that more intensive baseline treatment is associated with longer time to remission [31,32], the nonsignificant inverse association between immunosuppressive therapy and remission may reflect confounding by indication, whereby patients with more severe disease may require more aggressive treatment. With respect to sex, several retrospective cohorts have demonstrated that male patients with SLE tend to have higher disease activity and greater damage accrual [33,34]. Therefore, our observed association between female sex and a higher likelihood of achieving DORIS remission may reflect complex interactions among sex-related differences in disease phenotype, treatment strategies, and cohort characteristics. Additionally, LN diagnosis after 2016 was associated with a higher likelihood of achieving DORIS remission at 12 months. These findings may reflect evolving treatment paradigms, a greater emphasis on treat-to-target strategies and glucocorticoid minimization, differences in disease severity at presentation, and improvements in monitoring and longitudinal management. Because DORIS remission requires both effective control of disease activity and successful reduction of glucocorticoid exposure, such temporal changes in clinical practice may have contributed to the higher remission rates observed in more recent treatment eras.

Regarding renal response, baseline extrarenal involvement did not independently predict achievement of renal response at 12 months. Instead, nephrotic-range proteinuria, baseline eGFR and use of hydroxychloroquine were more strongly associated with treatment response. Higher baseline proteinuria and longer disease duration have been identified as negative predictors of renal response in LN [35], while baseline proteinuria of ≥2 g/day has been associated with a lower likelihood of achieving CRR in proliferative LN [36]. Consistent with these findings, nephrotic-range proteinuria in our cohort was associated with reduced odds of renal response. By contrast, use of hydroxychloroquine was strongly associated with a higher probability of achieving renal response, in line with previous reports demonstrating increased remission rates among patients receiving hydroxychloroquine [37].

Although both DORIS remission and renal response were assessed at 12 months, the predictors of these outcomes differed substantially. This divergence likely reflects the fact that the two outcomes capture distinct dimensions of disease control in LN. DORIS remission is a systemic treat-to-target endpoint requiring the absence of clinical disease activity and successful glucocorticoid tapering [27], and may therefore be influenced by overall SLE activity, systemic disease phenotype, serological activity, treatment intensity, and the ability to taper glucocorticoids [38]. In contrast, renal response is a kidney-specific outcome based primarily on improvement in proteinuria and preservation of renal function; it is more closely related to baseline renal disease burden, renal reserve, and histological severity on kidney biopsy [35]. Recent studies further demonstrate that systemic disease control and renal response provide complementary prognostic information: up to 44% of patients with renal response do not achieve LLDAS, while the attainment of both systemic disease control and renal response is associated with a lower risk of renal relapse [12,13]. Therefore, the non-overlapping predictors of DORIS remission and renal response are clinically plausible and suggest that systemic remission and renal response represent distinct yet complementary therapeutic targets in patients with LN.

This study has some limitations. First, the retrospective design and primary reliance on medical record data introduce the potential for selection bias, which may limit the generalizability of the findings. Second, we could not fully account for unmeasured confounding and temporal variations in treatment strategies. Future prospective studies are warranted to clarify the mechanisms linking extrarenal involvement and overall disease severity and to further refine risk stratification and therapeutic strategies for patients with SLE.

In conclusion, extrarenal involvement at the time of LN diagnosis was associated with higher baseline disease activity but did not independently predict 12-month renal response, achievement of DORIS remission, or long-term renal outcomes, including the risk of future renal flares. These findings suggest that, although extrarenal manifestations require careful monitoring and management due to their impact on systemic disease activity, they should not be considered independent determinants of prognosis in patients with SLE.

## Supporting information

S1 Table Multivariable Cox proportional hazards regression analyses for renal and extrarenal flares. (DOCX)

S2 Table Exploratory multivariable analysis of factors associated with DORIS remission at 12 months. (DOCX)

S3 Table Association of major and non-major extrarenal involvement with DORIS remission at 12 months. (DOCX)

S4 Table Association of major and non-major extrarenal involvement with renal response at 12 months. (DOCX)

S5 Table Exploratory multivariable analysis of factors associated with renal response at 12 months. (DOCX)
