# Supplementary material for: Prevalence and prognostic significance of extrarenal manifestations in patients with lupus nephritis: A retrospective cohort study
Source: PLoS One. 2026 Nov 10;21(11):e0362170. doi: 10.1371/journal.pone.0362170 (PMC13750565; doi:10.1371/journal.pone.0362170)
Supplement: Supplementary file 1 [file pone.0362170.s001.docx]

**Supplementary Table S1. Multivariable Cox proportional hazards regression analyses for renal and extrarenal flares**

|  | Renal flare, HR (95% CI) | P | Extrarenal flare, HR (95% CI) | P |
| --- | --- | --- | --- | --- |
| Age (per 10-year increase) | **1.154 (1.004–1.326)** | **0.044** | 1.794 (0.152**–**4.484) | 0.825 |
| Female | 1.082 (0.637**–**1.838) | 0.772 | 1.544 (0.856**–**2.785) | 0.149 |
| At baseline |  |  |  |  |
| Extrarenal involvement | 1.121 (0.678**–**1.853) | 0.657 | 0.964 (0.605**–**1.538) | 0.879 |
| SLEDAI (per 1-point increase) | 1.033 (0.978**–**1.091) | 0.247 | 1.002 (0.954**–**1.053) | 0.931 |
| Proliferative type LN | 0.811 (0.478**–**1.376) | 0.437 | 0.831 (0.516–1.338) | 0.831 |
| Nephrotic-range proteinuria | 1.212 (0.789**–**1.862) | 0.379 | 0.807 (0.492–1.323) | 0.395 |
| Medication |  |  |  |  |
| Hydroxychloroquine | 0.688 (0.365**–**1.220) | 0.668 | 1.274 (0.701**–**2.313) | 0.427 |
| IS drugs† | 1.288 (0.704**–**2.356) | 0.412 | 0.941 (0.594**–**1.490) | 0.795 |
| Belimumab | 1.740 (0.602–5.031) | 0.307 | **0.388 (0.175–0.860)** | **0.020** |
| Initial glucocorticoid dose | **1.113 (1.071–1.156)** | **0.001** | **1.130 (1.092–1.170)** | **0.001** |

†IS drugs include cyclophosphamide, mycophenolate mofetil, rituximab, and calcineurin inhibitors
