# Supplementary material for: Prevalence and prognostic significance of extrarenal manifestations in patients with lupus nephritis: A retrospective cohort study
Source: PLoS One. 2026 Nov 10;21(11):e0362170. doi: 10.1371/journal.pone.0362170 (PMC13750565; doi:10.1371/journal.pone.0362170)
Supplement: Supplementary file 2 [file pone.0362170.s002.docx]

**Supplementary Table S2. Exploratory multivariable analysis of factors associated with DORIS remission at 12 months**

|  | Univariable |  | Multivariable |  |
| --- | --- | --- | --- | --- |
|  | OR (95% CI) | *P* | OR (95% CI) | *P* |
| Age, per 10-year increase | 1.151 (0.613–2.162) | 0.662 | 0.995 (0.977–1.014) | 0.614 |
| BMI | **1.070 (1.001–1.145)** | **0.048** | 1.063 (0.986‒1.147) | 0.110 |
| Baseline eGFR | 0.999 (0.992–1.007) | 0.889 | 0.999 (0.990–1.007) | 0.766 |
| Female | **2.228 (1.279–3.879)** | **0.005** | **2.328 (1.248–4.341)** | **0.008** |
| Year of diagnosis |  |  |  |  |
| 2006‒2016 | - |  |  |  |
| 2016‒2022 | **1.822 (1.124–2.952)** | **0.015** | **2.004 (1.159–3.436)** | **0.013** |
| At baseline |  |  |  |  |
| Extrarenal involvement | 1.515 (0.928–2.474) | 0.097 | 1.042 (0.589‒1.843) | 0.888 |
| Mucocutaneous | 0.773 (0.323–1.854) | 0.565 |  |  |
| Constitutional | 0.718 (0.283–1.824) | 0.486 |  |  |
| Respiratory | 0.227 (0.029–1.770) | 0.157 |  |  |
| Cardiac | 2.197 (0.741–6.514) | 0.156 |  |  |
| Hematological | 0.786 (0.449–1.375) | 0.398 |  |  |
| SLEDAI (per 1-point increase) | **0.929 (0.886–0.973)** | **0.002** | **0.954 (0.902**‒**0.999)** | **0.048** |
| Proliferative type LN | 0.635 (0.390–1.036) | 0.069 |  |  |
| Nephrotic-range proteinuria | 1.092 (0.645–1.849) | 0.743 |  |  |
| aPL positivity† | 1.202 (0.739–1.957) | 0.458 |  |  |
| Medication |  |  |  |  |
| Hydroxychloroquine | 0.963 (0.533–1.740) | 0.901 |  |  |
| IS drugs‡ | **0.489 (0.301–0.795)** | **0.004** | 0.609 (0.356–1.041) | 0.070 |
| Belimumab | 1.782 (0.370–8.587) | 0.471 |  |  |
| Initial dose of GCs | 0.999 (0.998–1.001) | 0.313 |  |  |

†Antiphospholipid antibody (aPL) positivity was defined as positivity for at least one of the following: lupus anticoagulant, anti-β2 glycoprotein I antibody, or anticardiolipin antibody. ‡IS drugs include cyclophosphamide, mycophenolate mofetil, rituximab, and calcineurin inhibitors

LN: lupus nephritis; OR: odds ratio; IS drugs: immunosuppressive drugs; CI: confidence interval; SLEDAI**:** SLE Disease Activity Index; DORIS: Definition of Remission in SLE; BMI: body mass index; GCs: glucocorticoids
