# Supplementary material for: Prevalence and prognostic significance of extrarenal manifestations in patients with lupus nephritis: A retrospective cohort study
Source: PLoS One. 2026 Nov 10;21(11):e0362170. doi: 10.1371/journal.pone.0362170 (PMC13750565; doi:10.1371/journal.pone.0362170)
Supplement: Supplementary file 3 [file pone.0362170.s003.docx]

**Supplementary Table S3. Association of major and non-major extrarenal involvement with DORIS remission at 12 months**

|  | Univariable |  |
| --- | --- | --- |
|  | OR (95% CI) | *P* |
| Age (per 10-year increase) | 1.034 (0.888–1.205) | 0.664 |
| Female | **2.228 (1.279–3.879)** | **0.005** |
| At baseline |  |  |
| Extrarenal involvement | 1.515 (0.928–2.474) | 0.097 |
| Major organ involvement | 1.647 (0.771–3.516) | 0.197 |
| Non-major organ involvement | 1.148 (0.655–2.011) | 0.630 |
| SLEDAI (per 1-point increase) | **0.929 (0.886–0.973)** | **0.002** |
| Proliferative type LN | 0.635 (0.390–1.036) | 0.069 |
| Nephrotic-range proteinuria | 1.092 (0.645–1.849) | 0.743 |
| Medication |  |  |
| Hydroxychloroquine | 0.963 (0.533–1.740) | 0.901 |
| IS drugs† | **0.489 (0.301–0.795)** | **0.004** |
| Belimumab | 1.782 (0.370–8.587) | 0.471 |

†IS drugs include cyclophosphamide, mycophenolate mofetil, rituximab, and calcineurin inhibitors

LN: lupus nephritis; OR: odds ratio; IS drugs: immunosuppressive drugs; CI: confidence interval; SLEDAI**:** SLE Disease Activity Index; DORIS: Definition of Remission in SLE
