# Supplementary material for: Prevalence and prognostic significance of extrarenal manifestations in patients with lupus nephritis: A retrospective cohort study
Source: PLoS One. 2026 Nov 10;21(11):e0362170. doi: 10.1371/journal.pone.0362170 (PMC13750565; doi:10.1371/journal.pone.0362170)
Supplement: Supplementary file 4 [file pone.0362170.s004.docx]

**Supplementary Table S4. Association of major and non-major extrarenal involvement with renal response at 12 months**

|  | Univariable |  |
| --- | --- | --- |
|  | OR (95% CI) | *P* |
| Age (per 10-year increase) | **0.817 (0.700–0.954)** | **0.011** |
| Female | 1.408 (0.800–2.479) | 0.235 |
| At baseline |  |  |
| Extrarenal involvement | 0.823 (0.508–1.333) | 0.429 |
| Major organ involvement | 1.162 (0.578–2.335) | 0.673 |
| Non-major organ involvement | 1.045 (0.594–1.838) | 0.878 |
| SLEDAI (per 1-point increase) | 1.015 (0.959–1.074) | 0.608 |
| Proliferative type LN | 1.364 (0.846–2.200) | 0.202 |
| Nephrotic-range proteinuria | **0.509 (0.280–0.924)** | **0.027** |
| Medication |  |  |
| Hydroxychloroquine | **4.630 (2.655–8.076)** | **<0.001** |
| IS drugs† | 0.967 (0.660–1.560) | 0.891 |
| Belimumab | 3.777 (0.474–30.102) | 0.210 |

†IS drugs include cyclophosphamide, mycophenolate mofetil, rituximab, and calcineurin inhibitors

LN: lupus nephritis; OR: odds ratio; IS drug: immunosuppressive drugs; CI: confidence interval; SLEDAI**:** SLE Disease Activity Index
