# Supplementary material for: Prevalence and prognostic significance of extrarenal manifestations in patients with lupus nephritis: A retrospective cohort study
Source: PLoS One. 2026 Nov 10;21(11):e0362170. doi: 10.1371/journal.pone.0362170 (PMC13750565; doi:10.1371/journal.pone.0362170)
Supplement: Supplementary file 5 [file pone.0362170.s005.docx]

**Supplementary Table S5. Exploratory multivariable analysis of factors associated with renal response at 12 months**

|  | Univariable |  | Multivariable |  |
| --- | --- | --- | --- | --- |
|  | OR (95% CI) | *P* | OR (95% CI) | *P* |
| Age, per 10-year increase | **0.507 (0.282–0.911)** | **0.023** | 0.990 (0.971–1.009) | 0.289 |
| BMI | 0.991 (0.913–1.076) | 0.831 | 0.982 (0.907‒1.062) | 0.644 |
| Baseline eGFR | **1.027 (1.019‒1.035)** | **<0.001** | **1.026 (1.017‒1.034)** | **<0.001** |
| Female | 1.408 (0.800–2.479) | 0.235 | 1.437 (0.742‒2.785) | 0.282 |
| Year of diagnosis |  |  |  |  |
| 2006‒2016 | - |  |  |  |
| 2016‒2022 | 1.326 (0.810–2.170) | 0.262 | 1.190 (0.677–2.093) | 0.546 |
| At baseline |  |  |  |  |
| Extrarenal involvement | 0.823 (0.508–1.333) | 0.429 | 0.930 (0.515–1.679) | 0.809 |
| Mucocutaneous | 1.071 (0.456–2.515) | 0.875 |  |  |
| Constitutional | 0.910 (0.376–2.202) | 0.834 |  |  |
| Respiratory | 0.405 (0.132–1.244) | 0.114 |  |  |
| Cardiac | 1.424 (0.380–5.341) | 0.600 |  |  |
| Hematological | 0.965 (0.552–1.685) | 0.900 |  |  |
| SLEDAI (per 1-point increase) | 1.015 (0.959–1.074) | 0.608 |  |  |
| Proliferative type LN | 1.364 (0.846–2.200) | 0.202 |  |  |
| Nephrotic-range proteinuria | **0.509 (0.280–0.924)** | **0.027** | **0.811 (0.432–0.974)** | **0.045** |
| aPL positivity† | 1.325 (0.804‒2.183) | 0.710 | 1.059 (0.600‒1.869) | 0.843 |
| Medication |  |  |  |  |
| Hydroxychloroquine | **4.630 (2.655–8.076)** | **<0.001** | **4.294 (2.411–7.649)** | **<0.001** |
| IS drugs‡ | 0.967 (0.660–1.560) | 0.891 |  |  |
| Belimumab | 3.777 (0.474–30.102) | 0.210 |  |  |
| Initial dose of GCs | 0.999 (0.998–1.000) | 0.086 |  |  |

Renal response was defined as achievement of either complete renal response or partial renal response at 12 months after LN diagnosis. †Antiphospholipid antibody (aPL) positivity was defined as positivity for at least one of the following: lupus anticoagulant, anti-β2 glycoprotein I antibody, or anticardiolipin antibody. ‡IS drugs include cyclophosphamide, mycophenolate mofetil, rituximab, and calcineurin inhibitors

LN: lupus nephritis; OR: odds ratio; IS drugs: immunosuppressive drugs; CI: confidence interval; SLEDAI**:** SLE Disease Activity Index; BMI: body mass index; eGFR: estimated glomerular filtration rate; aPL: antiphospholipid antibody; GCs: glucocorticoids
